# Decision-Making for Space Maintenance After the Premature Loss of Primary First Molars: A Clinically Oriented Narrative Review

**DOI:** 10.7759/cureus.104764

**Published:** 2026-03-06

**Authors:** Gopal Babu, Sindhuja Arunachalam, Garima Jindal, Ankitu Sahai, Ritambhra Dikshit, Shahina Khanam, Tanya Pandey

**Affiliations:** 1 Pediatric Dentistry, King George's Medical University, Lucknow, IND

**Keywords:** decision-making, premature loss, primary first molar, space maintenance, space management

## Abstract

The aim of this narrative review is to critically integrate the available evidence on space changes following the premature loss of primary first molars and to develop a clinically orientated, evidence-based decision-making framework for space maintenance. A clinically focused narrative review was conducted using PubMed and Google Scholar databases. Studies that met the eligibility criteria and were published in the English language between January 1998 and December 2025 were included. Fourteen primary studies and six systematic reviews were selected based on PECO (population, exposure, control, and outcome) criteria. Evidence was synthesized narratively, rather than through a quantitative meta-analysis, due to substantial heterogeneity. Eruption and stable intercuspation of the first permanent molar, as well as a leptoprosopic facial growth pattern, consistently emerged as independent predictors of clinically relevant space loss. In contrast, archetype, molar relationship, time elapsed since extraction, and crowding functioned primarily as modifying factors. Clinical decision-making should be individualized using a structured, risk-based framework that integrates biological and occlusal determinants rather than relying solely on tooth loss.

## Introduction and background

A range of environmental and morphogenic factors influences occlusal development. Crucially, the presence of primary teeth is vital. Their eventual physiological exfoliation facilitates alveolar growth, which in turn ensures sufficient space for the successful eruption and alignment of the permanent dentition.

The prevalence of early loss of primary teeth ranges widely, from 5.8% to 65.4% [1]. Premature loss of primary teeth (PLPT) is defined as the loss of a primary tooth one year before the expected chronological period for physiological exfoliation or while the successor permanent tooth is still before Nolla's stage 6, evidenced through radiographic examination, where the coronal formation is complete. The root is less than two-thirds completed [2,3]. This decreases the arch length required for permanent teeth, exacerbating the patient's malocclusion, crowding, rotation, ectopic eruption, extraction of the antagonist tooth, crossbite, increased overjet and overbite, inappropriate molar relationship, craniofacial growth disturbances, and impaction of permanent teeth in particular [4,5].

Therefore, it has been suggested that maintaining primary teeth during the transition from primary to full permanent dentition is essential to minimize space loss and reduce the need for, or the complexity of, orthodontic management [6,7]. There is a broad consensus on the need for a space maintainer after the loss of a primary second molar; however, the need for space maintenance after the early loss of a primary first molar remains highly debated.

Zhao et al. conducted a comprehensive meta-analysis of split-mouth studies, confirming that while measurable space loss occurs after premature loss of primary first molars, arch width, arch length, and arch perimeter remain largely unaffected over medium-term follow-up. Factors such as age, time since tooth extraction, facial pattern, and molar relationships influence the space change after the premature loss of primary first molars [8].

Existing systematic reviews, meta-analyses, and longitudinal studies quantify space loss and stress the need for individualized space management following premature primary first molar loss, based on a comprehensive assessment of various characteristics [9,10]. However, this evidence does not translate into a stepwise, clinically applicable decision pathway that integrates eruption status, facial growth, extraction timing, the arch involved, and occlusal modifiers. Due to the high heterogeneity in studies, clinicians must rely on subjective judgment rather than evidence-linked criteria.

Thus, this review aims to address this gap by synthesizing biological, occlusal, and growth determinants identified across systematic reviews and primary studies into a structured decision-making framework to support consistent, evidence-informed chairside management, moving beyond descriptive statistics to develop the structured, risk-based decision-making framework proposed in this study.

## Review

Search strategy

A targeted electronic search was conducted using the PubMed and Google Scholar databases to identify relevant English-language publications from 1998 to 2025. The keywords used in the search were premature loss of primary first molars, spatial changes, space loss, space changes, space maintainer, and space maintenance. Additionally, the reference lists of all included studies and pertinent systematic reviews were manually searched to identify any additional eligible articles. The PECO (population, exposure, control, and outcome) framework was used to guide the search.

The population included children in the primary and mixed dentition periods. The exposure considered was the premature loss (either unilateral or bilateral) of primary first molars in the maxillary and/or mandibular arches. The comparison consisted of either the contralateral arch without tooth loss or studies in which no explicit control group was present. The outcomes assessed included the magnitude and pattern of space loss, changes in arch dimensions, the clinical relevance of these space changes, and whether these findings indicate the need for placement of a space maintainer.

This is a clinically oriented narrative review. The search strategy was purposeful rather than exhaustive, as it aimed at identifying clinically relevant evidence. Formal risk-of-bias assessment and quantitative synthesis were not performed.

Eligibility criteria

Inclusion Criteria

Studies were included if they met the following criteria: (1) included children in the primary or mixed dentition; (2) evaluated premature loss of primary first molars in the maxillary and/or mandibular arches; (3) reported outcomes related to space loss, arch dimensional changes, tooth movement, angulation changes, or orthodontic implications; (4) published as original research articles, observational or longitudinal studies, split-mouth studies, cohort or cross-sectional studies, clinical trials, systematic reviews, or meta-analyses; and (5) available as full-text articles in English.

Exclusion Criteria

Studies were excluded if they were (1) case reports, case series, expert opinions, editorials, or narrative reviews without a structured methodology; (2) animal studies, in vitro studies, or experimental laboratory-based research; (3) studies focused on primary second molars or permanent teeth exclusively, without separate or extractable data for primary first molars; (4) studies conducted solely in adult populations; (5) publications lacking clearly defined space-related outcomes; and (6) conference abstracts, theses, or unpublished literature where full methodological details were unavailable.

Study selection and data extraction

Titles and abstracts were screened for relevance. Full texts of potentially eligible articles were subsequently reviewed to confirm inclusion. Study quality was implicitly weighted based on design.

Finally, the study included 14 primary studies and six systematic reviews. Data were charted using a standardized extraction framework. They included author and year, study design, arch involved, sample size, measurement methods, follow-up duration, and principal findings related to space changes and space management.

Synthesis of results

**Figure 1:**
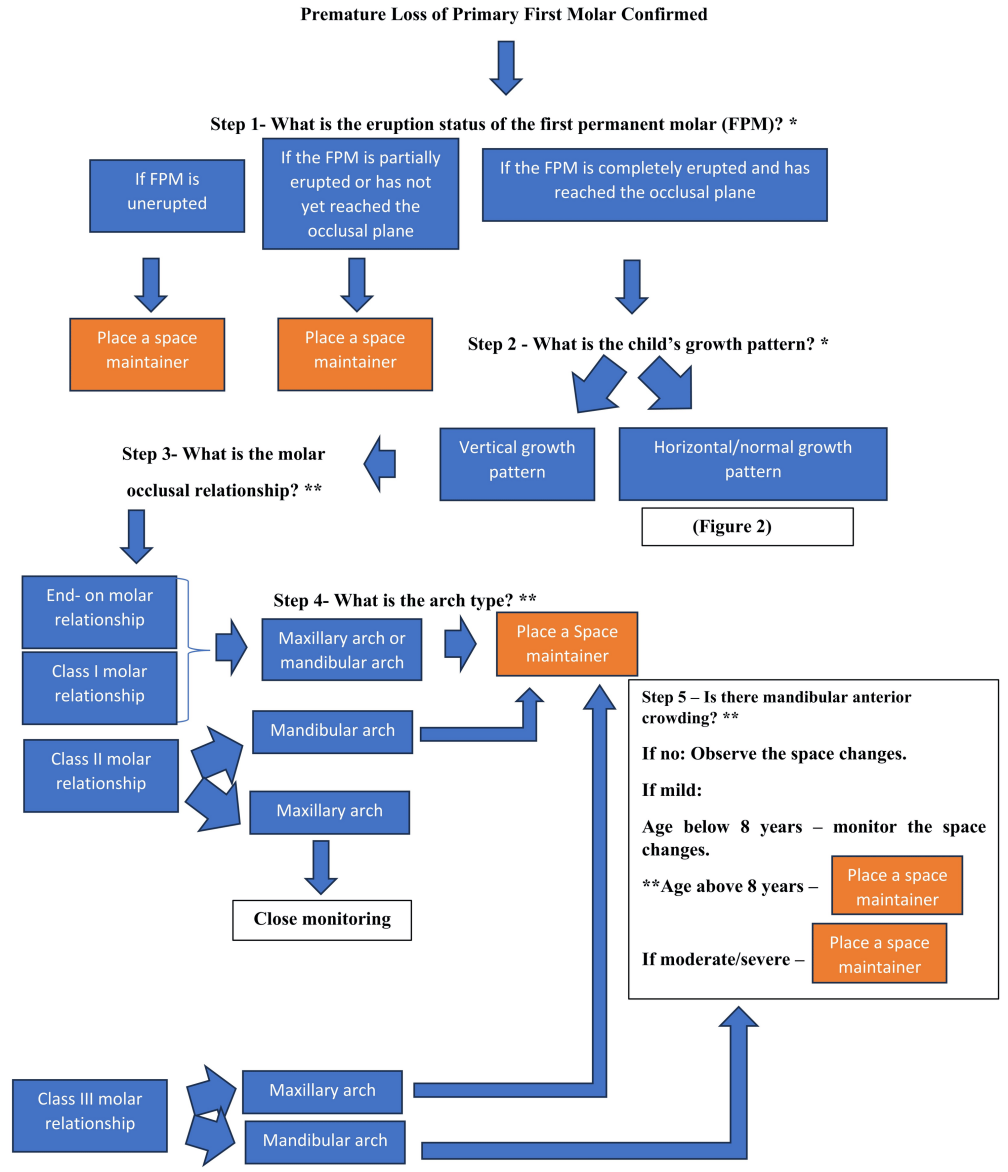
Evidence-based clinical decision-making algorithm for space maintenance following premature loss of primary first molars *: independent predictors; **: modifiers

**Figure 2:**
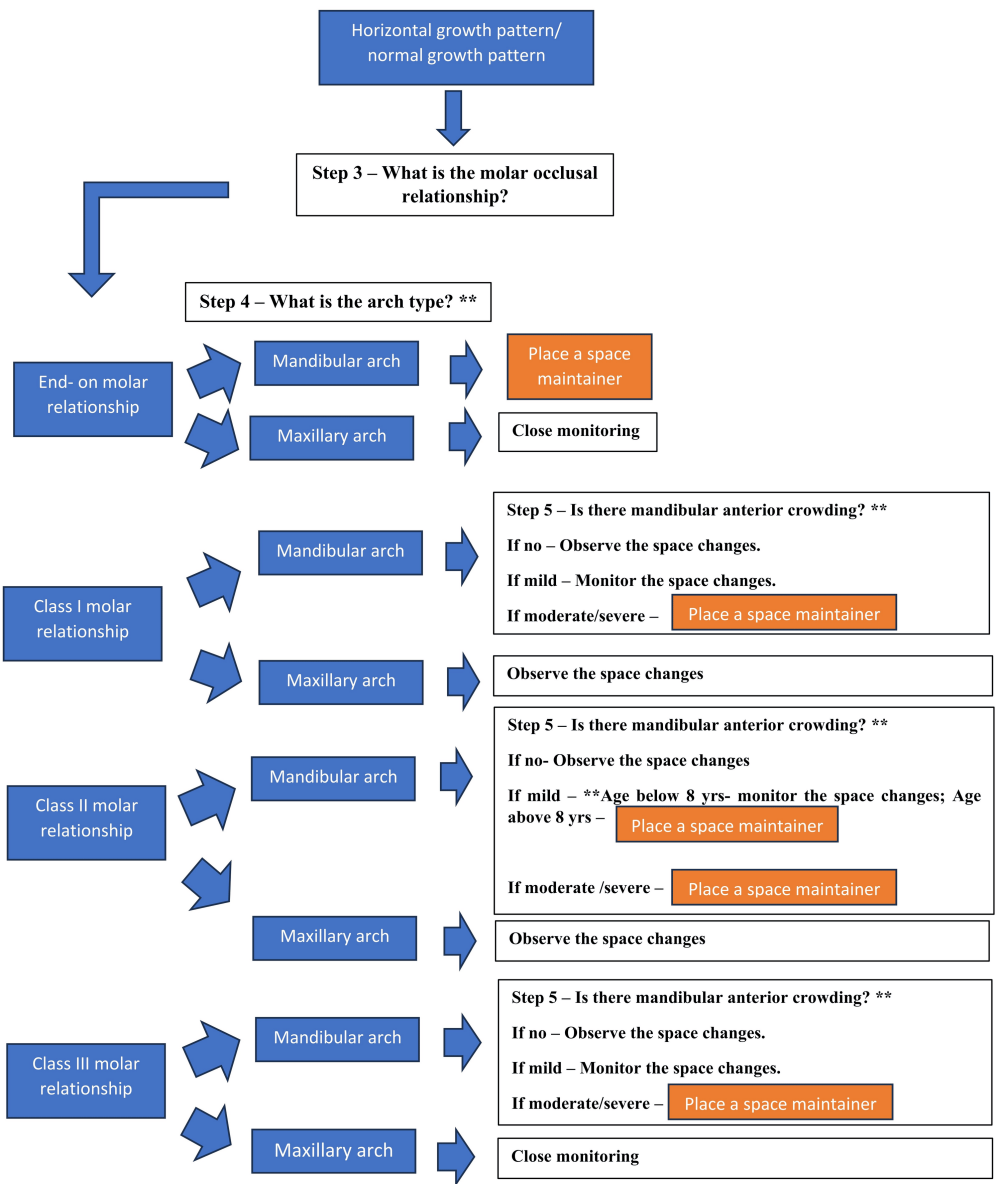
Evidence-based clinical decision-making algorithm for space maintenance following premature loss of primary first molars (continued) *: independent predictors; **: modifiers

Due to substantial heterogeneity in study designs, outcome measures, and follow-up periods, among other factors, a quantitative assessment was not performed. The findings were synthesized narratively, with an emphasis on patterns of space change, arch-specific differences, adjacent tooth drift, modifying factors, and clinical implications for space maintenance following the premature loss of primary first molars. A decision-making flowchart was framed to guide clinicians, as shown in Figures 1-2.

Discussion

The findings are presented as a factor-wise synthesis, moving beyond isolated measurements of space loss to evaluate which variables consistently modify the magnitude, direction, and clinical significance of post-extraction changes. This approach allows differentiation between independent predictors, modifying factors, and non-determinant variables, thereby providing a clinically meaningful framework for decision-making rather than a purely descriptive summary of dimensional changes.

Independent Predictors of Space Loss

Eruption status of the first permanent molar and facial growth pattern were identified in the literature as "high-order determinants" that consistently predict whether clinically relevant space loss will occur, regardless of other co-existing variables, thus classified as independent predictors.

Influence of the Eruption Status of the First Permanent Molar on Space Loss

Space maintenance in the mixed dentition is not as crucial as it is in the primary dentition, because the permanent first molar has already erupted. Several authors demonstrated clinically insignificant space loss and significantly less tooth displacement (in canines and second primary molars), suggesting that no active intervention was necessary unless leeway space was to be preserved [11-15]. This is because, after the eruption of the first permanent molar, it is almost passive, thereby not producing a mesial component of the eruption force on the adjacent teeth. This recommendation was also supported by the American Academy of Pediatric Dentistry [16].

Although all these studies specified that they included fully erupted first permanent molars with complete intercuspation, most did not mention the occlusal relationship. Alexander et al. found that the end-on molar relationship, regardless of the growth pattern, resulted in statistically significant space loss in the mandibular arch. They demonstrated that when the first permanent molar was in an end-to-end relationship before an early or late mesial shift had occurred, space loss may occur because of potential molar occlusal adjustments [17].

Thus, the eruption of the first permanent molar alone does not suffice for occlusal stability; however, interdigitation and intercuspation play a critical role in determining post-extraction space behavior. Therefore, a fully erupted and intercuspated first permanent molar (Step 1) substantially reduces the need for space maintenance, supporting observation rather than routine appliance therapy in such cases. However, in cases where unfavorable intercuspation is present, an individualized approach should be followed for space maintenance.

Facial Growth Pattern

Facial morphology has emerged as a high-order determinant of space loss (Step 2). Multivariate analyses by Mosharrafian et al. and Heidari et al. identified the leptoprosopic facial pattern as a significant independent predictor of greater space loss in both arches [18,15]. Alexander et al. corroborated this, reporting statistically significant space loss in leptoprosopic individuals regardless of molar relationship. This susceptibility is attributed to weaker musculature and reduced tonicity in long-faced individuals, which facilitates easier orthodontic tooth movement [17]. Conversely, mesoprosopic and euryprosopic patterns appear protective, often maintaining arch integrity even in the presence of minor space loss.

Drifting of Adjacent Teeth

Current evidence regarding the movement of adjacent teeth challenges the traditional assumption that space loss is driven solely by mesial molar migration. Lin and Chang and Lin et al. indicated that early space changes are predominantly attributable to the distal drift of the primary canine in the mandible and a combination of distal canine drift and palatal incisor migration in the maxilla [19,20]. While theoretically attributed to perioral and mentalis muscle pressure, the observed stability of the inter-canine arch length suggests these movements are subtle adjustments rather than extensive drifts.

Heidari et al. quantified this mechanism using contact-point measurements, demonstrating that while distal canine movement contributed more to space reduction than posterior movement in approximately 23% of cases, the magnitude was clinically insignificant (0.2-0.7 mm) and insufficient to independently justify space maintenance [15].

Three-dimensional analyses further corroborate the lack of substantial collapse. Kim et al. and Park et al. reported no significant changes in canine inclination, observing only minor mesial tipping of mandibular posterior teeth [21,11]. Conversely, in the maxilla, studies by Kim et al. and Cernei et al. documented distal rather than mesial angulation of permanent molars, a phenomenon attributed to the eruptive spurts of the first premolar exerting a distalizing influence [21,22].

Overall, available evidence concludes that accurate tooth movement after primary first molar loss does not follow the uniform distal canine drift or mesial molar drift. This aligns with Kronfeld's theory, where the neutral zone is situated between the bicuspids in the mandible and mesial to the molar in the maxilla. The teeth anterior to this zone drift posteriorly, and the posterior ones drift anteriorly [23]. The current evidence suggests that post-extraction space loss results from a complex interplay of minor anterior and posterior movements rather than a single dominant mechanism. The magnitude of these movements is typically below thresholds of clinical relevance.

Modifying Factors

These variables influence the magnitude or pattern of space loss but do not, in isolation, justify the placement of a space maintainer. They serve to refine the clinical management strategy (e.g., observation vs. close monitoring) rather than dictating the intervention itself.

Molar Occlusal Relationship

Alexander et al. identified the end-on molar relationship as a critical vulnerability; in these cases, the physiological drive for a late mesial shift to achieve Class I occlusion can precipitate space loss, particularly in the mandible, even in mesoprosopic individuals who would otherwise be stable. Conversely, a fully interdigitated Class I occlusion acts as a mechanical "lock," offering greater resistance to mesial drift [17]. Additionally, Heidari et al. observed that the specific pattern of loss correlates with malocclusion type, with Class II relationships predisposing to greater mandibular space loss and Class III relationships favoring maxillary loss [15]. Consequently, while the molar relationship does not automatically trigger intervention, the presence of an end-on relationship warrants heightened vigilance compared to a stable Class I intercuspation. Thus, the molar occlusal relationship functions as a modifying factor (Step 3).

Dental Arch Dimensions and Patterns of Space Loss

Integrated findings from longitudinal and three-dimensional studies reveal a critical distinction between localized space reduction and arch collapse. While space loss occurs, it follows a consistent, arch-specific pattern that rarely compromises overall dimensional stability.

Evidence consistently identifies the mandibular arch as significantly more susceptible to space loss than the maxillary arch. Longitudinal studies by Lin and Chang, Lin et al., Padma Kumari and Retnakumari, and Mosharrafian et al. demonstrate that while the mandible undergoes statistically significant and progressive space reduction on the extraction side, the maxillary arch typically exhibits only minimal loss (approximately 1 mm) that stabilizes quickly [19,12,24,18]. This disparity is biomechanically attributed to the mandible's larger leeway space and the dominance of centripetal forces, where lower lip pressure (11.6 g/cm²) exceeds tongue pressure (10 g/cm²) [25]. Conversely, the maxilla's stability is often reinforced by the divergent, labial eruption path of the permanent successors.

Crucially, localized space loss does not inevitably translate into global arch collapse. Classic 8-12-month longitudinal analyses [13,14,19,24] and recent meta-analyses [8] uniformly report that despite a reduction in the extraction site, arch width, arch length, and arch perimeter remain largely unaffected. Long-term follow-up by Lin and Lin even demonstrated a significant increase in maxillary arch dimensions over 81 months, suggesting that physiologic growth and dentoalveolar compensation often override the effects of premature loss [26].

Discrepancies in the literature regarding the magnitude of loss are largely methodological. While traditional 2D caliper studies often report "no change" due to measurement limitations, high-precision 3D studies detect minor "physiologic drifts" and tipping movements [21,11,27]. However, even when 3D analyses identify statistically significant reductions as seen in Kundra et al.'s recent findings, these changes often represent minor angulation adjustments rather than skeletal constriction [27].

Consequently, arch type functions as a modifying factor (Step 4) rather than an independent predictor of space loss. The evidence suggests that the fear of arch collapse does not justify routine space maintenance. Instead, the arch involved dictates the intensity of monitoring: mandibular loss warrants closer surveillance due to higher susceptibility, whereas maxillary loss is generally compatible with observation unless compounded by other risk factors.

Influence of Crowding and Midline Deviation

Space loss is greater following premature tooth loss in patients with dental crowding compared with those with dental spacing. Padma Kumari and Retnakumari discussed that large dental arches with normal or excessive space are not affected by early loss of primary teeth. In contrast, in arches with pre-existing crowding or reduced available space, leeway space is more readily consumed by adjacent tooth movement [24].

In contrast, Mosharrafian et al. and Heidari et al. found, in their multivariate analysis, that crowding had no significant effect on space loss [18,15]. However, these findings should be interpreted cautiously, given the limited representation of severely crowded cases. According to Moorrees and Reed, if crowding has already developed in the mandible by age 8, it will not resolve with continued growth and development, as the inter-canine arch width would have already been stabilized; even a negligible space loss becomes clinically significant [28].

Earlier reports suggest midline deviation due to the distal deviation of the canine as a result of unilateral premature loss of a deciduous first molar in crowded arches [29]. However, recent multivariate regression analyses by Heidari et al. reported no midline deviation in most cases. But this cannot be considered valid, as there was only one case with severe crowding [15].

To sum up, pre-existing crowding (particularly in the mandibular arch and among older children) may act as a modifier (Step 5) of space loss, reducing leeway space and magnifying the clinical impact of even minimal space loss, rather than being an independent factor.

Time Elapsed Since Tooth Loss

A consistent finding across numerous observational, longitudinal, and three-dimensional studies is that space loss after the premature extraction of primary first molars is highly time-dependent, with the most significant tooth movement occurring early after extraction. Lin et al., Lin et al., and Padma Kumari and Retnakumari reported rapid space reduction within the first 4-6 months, with only minimal additional space loss occurring between months 6 and 12, indicating a clear deceleration and plateauing of space closure [20,12,24]. This early vulnerability corresponds to a period of tooth mobility driven by eruptive forces, incomplete intercuspation, and occlusal stabilization, after which adjacent teeth achieve positional equilibrium.

Cross-sectional studies by Mosharrafian et al. reported increasing space loss 6-9 months after tooth extraction, while Heidari et al. found greater space loss at 8-18 months post-extraction [18,15]. This can be explained by the extraction performed during the early mixed dentition period, when active eruption had increased space loss. Increased bone density, reduced eruptive forces, and established intercuspation in late mixed dentition limit further tooth movement. The observed changes, therefore, represent cumulative early loss rather than increased movement occurring in later periods.

Accordingly, the time elapsed should be regarded as a modifying factor operative during early mixed dentition. It should not, in and of itself, justify the placement of a space maintainer in late mixed dentition.

Statistically Significant Space Loss Versus Clinically Significant Space Loss

Even when space loss occurs, as shown in the selected studies, the magnitude of the loss must be taken into account. Zhao et al. reported that over a follow-up period of 6-24 months, statistically, space loss was 0.65 mm for the maxillary D+E, 1.24 mm for the mandibular D+E, and 1.47 mm for the mandibular D [8]. However, this magnitude of loss generally falls within the physiological leeway space and therefore may not compromise the eruption of the permanent successors in otherwise well-aligned arches.

However, in specific clinical situations, this amount of space loss could have treatment implications, such as crowding, incisor or lip protrusion, or an accentuated curve of Spee. Even a 3-mm reduction in arch length (approximately 1.5 mm per side) can alter orthodontic decision-making and increase the likelihood of requiring extraction therapy. However, the actual clinical relevance of any space loss can be judged only after a detailed evaluation of the patient's occlusal condition and a comprehensive assessment of all variables influencing arch space. Therefore, the clinical importance of space reduction cannot be universally applied to all cases and should be determined on an individual basis for each patient [30].

Evidence Synthesis From Systematic Reviews

Across systematic reviews [8-10,30-32], premature loss of a primary first molar is consistently associated with early, localized space loss that is generally small and clinically variable. The loss occurs mainly within the first few months after extraction, is more pronounced in the mandibular arch than in the maxillary arch, and is primarily due to drift of adjacent teeth rather than generalized arch collapse. Notably, most reviews report no clinically significant changes in overall arch width, arch length, or arch perimeter. The clinical relevance of the observed space loss is inconsistent and depends on modifying factors such as pre-existing crowding, facial pattern, occlusal relationships, and timing of extraction. Consequently, the evidence does not support routine space maintainer placement following premature loss of a primary first molar, favoring individualized, case-specific decision-making.

Although all included systematic reviews consistently advocate an individualized, case-based approach following premature loss of a primary first molar, none provide a structured or operational framework to guide clinicians in deciding whether to place or withhold a space maintainer.

Tables 1-2 present the key characteristics and main conclusions of the included primary studies and systematic reviews.

**Table 1:** Key characteristics and main conclusions of the included primary studies D+E space: space between the distal surface of the primary canine and the mesial surface of the primary second molar; 3D: three-dimensional; CT: computed tomography

Author (year)	Study design	Sample size (n)	Arch evaluated	Method of measurement	Follow-up	Principal findings
Park et al. (2009) [11]	Prospective longitudinal split-mouth study	11 children	Maxilla	3D digital model analysis	12 months	No significant space loss compared with control (p=0.33); arch dimensions increased with normal growth
Lin et al. (2011) [12]	Prospective longitudinal split-mouth study	13 children	Maxilla	Cast measurements	12 months	Approximately 1 mm space loss (p<0.05); no significant molar tipping or arch dimensional changes
Macena et al. (2011) [13]	Prospective longitudinal split-mouth study	20 children	Maxilla and mandible	Cast, clinical, and radiographic assessment	10 months	No statistically significant reduction in extraction space, arch length, or hemi-perimeter; adjacent tooth position stable
Kobylińska et al. (2019) [14]	Prospective observational split-mouth study	27 children	Maxilla and mandible	Cast measurements	12 months	Significant interdental space reduction (0.9-1 mm); no clinically relevant restriction of permanent tooth eruption
Heidari et al. (2022) [15]	Cross-sectional observational study with regression analysis	47 children	Maxilla and mandible	Cast measurements	≥6 months post-extraction	Mean space loss 0.56 mm (maxilla 0.54 mm; mandible 0.58 mm); not clinically significant; facial pattern, molar relationship, and extraction timing influenced space loss
Alexander et al. (2015) [17]	Prospective split-mouth observational study	226 children	Maxilla and mandible	Cast measurements	9 months	Significant space loss in leptoprosopic facial pattern (≈0.9-1.8 mm); facial pattern and molar relationship influenced the magnitude of space loss
Mosharrafian et al. (2021) [18]	Cross-sectional split-mouth study with regression analysis	50 children	Maxilla and mandible	Cast measurements	≥6 months post-extraction	Mean space loss 1.36±0.78 mm; mandibular loss greater than maxillary; facial pattern and time since extraction were significant predictors
Lin and Change (1998) [19]	Prospective longitudinal split-mouth study	21 children	Mandible	Cast measurements with digital calipers	8 months	Significant reduction in D+E space on extraction side compared with control (p=0.025); no significant changes in arch width, arch length, or arch perimeter
Lin et al. (2007) [20]	Prospective longitudinal split-mouth study	19 children	Maxilla	Cast measurements	6 months	Mean space loss approximately 1 mm (p<0.01); no mesial movement of permanent molars; arch dimensions remained stable
Kim et al. (2008) [21]	Prospective longitudinal observational study	6 children	Maxilla and mandible	3D laser-scanned digital models	Not specified	No measurable maxillary space loss; mandibular space loss observed in two of three cases; arch width and perimeter remained stable
Cernei et al. (2015) [22]	Cross-sectional radiographic comparative study	64 children	Maxilla	Panoramic radiographic analysis	Not specified	Mild distal angulation of permanent molars following premature loss; angular changes clinically limited
Padma Kumari and Retnakumari (2006) [24]	Prospective longitudinal clinical study	40 children	Mandible	Cast measurements	12 months	Significant space loss on extraction side (p<0.01), greatest within the first four months; no significant changes in arch width, length, or perimeter
Lin and Lin (2017) [26]	Long-term prospective split-mouth study	9 children	Maxilla	Cast measurements	81 months	No significant crowding or canine impaction; significant increases in arch width and arch length with growth (p<0.05)
Kundra et al. (2024) [27]	Longitudinal split-mouth study	20 children	Maxilla and mandible	3D CT (Dentascan) analysis	9 months	Significant reduction in arch length, hemi-perimeter, and D-space in both arches; mandibular changes more pronounced

**Table 2:** Key characteristics and main conclusions of the included systematic reviews D+E space: space between the distal surface of the primary canine and the mesial surface of the primary second molar; 3D: three-dimensional; CT: computed tomography; PRISMA: Preferred Reporting Items for Systematic Reviews and Meta-Analyses; ROBINS-I: Risk Of Bias In Non-randomized Studies - of Interventions; PROSPERO: International Prospective Register of Systematic Reviews; ADA: American Dental Association

Author (year)	Study design	Number of studies included	Total sample size	Arch evaluated	Main outcomes assessed	Principal conclusions
Zhao et al. (2023) [8]	Systematic review and meta-analysis (PRISMA; ROBINS-I assessed)	8 studies	477 children	Maxilla and mandible	D+E space loss, arch width, arch length, arch perimeter	Mean space loss was 0.65 mm in the maxilla and 1.24-1.47 mm in the mandible; space loss occurred early and stabilized; no significant changes in arch width, length, or perimeter; facial pattern, age, and extraction timing influenced space loss
Gibas-Stanek and Loster (2018) [9]	Systematic review	20 studies	>1,000 children	Maxilla and mandible	Space loss, arch changes, malocclusion development	Maxillary loss associated mainly with distal canine drift; mandibular loss showed greater and less predictable space loss; clinical impact modified by age, facial pattern, crowding, and molar relationship
Kaklamanos et al. (2017) [10]	Systematic review and meta-analysis (PRISMA; ADA risk-of-bias assessment)	2 controlled split-mouth studies	51 children	Mandible	Space loss over time	Progressive mandibular space loss observed, reaching mean loss of approximately 1.5 mm at eight months; clinical relevance dependent on individual occlusal factors
Tunison et al. (2008) [30]	Systematic review of controlled longitudinal studies	6 studies	80 children	Maxilla and mandible	Space loss, arch width, arch length, arch perimeter	Mean space loss approximately 1 mm in the maxilla and 1.5 mm in the mandible; arch dimensions generally unaffected; space maintainer indicated selectively in patients with crowding or arch length deficiency
Joshi et al. (2025) [31]	Systematic review and meta-analysis (PRISMA; PROSPERO-registered)	7 studies	120 children	Maxilla	D+E space loss, arch dimensions, need for space maintainer	Significant extraction site space loss observed; however, arch width, length, and perimeter remained stable; modifying factors included age, facial pattern, and molar relationship
Gandhi and Gurunathan (2022) [32]	Systematic review (PRISMA; PROSPERO-registered)	6 first molar-specific studies	Approximately 105 children	Maxilla and mandible	Short- and long-term space loss and mechanisms	Early space loss primarily due to distal canine migration; mandibular loss greater than maxillary; arch dimensions generally maintained over long-term follow-up

Methodological Limitations

This review acknowledges several limitations inherent to its clinically oriented narrative design. Unlike a systematic review, the search strategy was purposeful rather than exhaustive, and a formal risk-of-bias assessment was not conducted, potentially introducing selection bias as study quality was implicitly rather than explicitly weighted. Furthermore, a quantitative meta-analysis was not feasible due to substantial heterogeneity across included studies regarding follow-up durations, patient age groups, and measurement techniques, which ranged from manual calliper measurements on two-dimensional stone casts to high-precision three-dimensional digital analysis. Consequently, the findings represent a qualitative factor-wise synthesis rather than a statistical aggregation; while this approach permits the identification of high-order determinants such as facial growth patterns and eruption status, it precludes the definitive statistical quantification of the specific weight each variable contributes to space loss.

## Conclusions

This review demonstrates that eruption and stable intercuspation of the first permanent molar and facial growth pattern, particularly a leptoprosopic morphology, emerge as the most consistent independent predictors of clinically relevant space loss, whereas factors such as molar relationship, arch involved, time elapsed since extraction, and crowding function primarily act as modifying or contextual variables rather than standalone indications for space maintainer placement. By integrating evidence from longitudinal, split-mouth, cross-sectional, and three-dimensional studies, this review moves beyond isolated measurements of space loss. It proposes a clinically orientated, decision-linked framework to guide space management. This framework emphasizes risk stratification over tooth-based assumptions and supports individualized treatment planning based on biological and occlusal determinants rather than extraction status alone.
